# Bifurcation analysis of motoneuronal excitability mechanisms under normal and ALS conditions

**DOI:** 10.3389/fncel.2023.1093199

**Published:** 2023-02-16

**Authors:** Muhammad Moustafa, Mohamed H. Mousa, Mohamed S. Saad, Tamer Basha, Sherif M. Elbasiouny

**Affiliations:** ^1^Department of Systems and Biomedical Engineering, Faculty of Engineering, Cairo University, Giza, Egypt; ^2^Department of Biomedical, Industrial, and Human Factors Engineering, College of Engineering and Computer Science, Wright State University, Dayton, OH, United States; ^3^Department of Electrical Power Engineering, Faculty of Engineering, Cairo University, Giza, Egypt; ^4^Department of Neuroscience, Cell Biology and Physiology, Boonshoft School of Medicine and College of Science and Mathematics, Wright State University, Dayton, OH, United States

**Keywords:** ALS, bifurcation, excitability, motoneuron, modeling, XPPAUT

## Abstract

**Introduction:**

Bifurcation analysis allows the examination of steady-state, non-linear dynamics of neurons and their effects on cell firing, yet its usage in neuroscience is limited to single-compartment models of highly reduced states. This is primarily due to the difficulty in developing high-fidelity neuronal models with 3D anatomy and multiple ion channels in XPPAUT, the primary bifurcation analysis software in neuroscience.

**Methods:**

To facilitate bifurcation analysis of high-fidelity neuronal models under normal and disease conditions, we developed a multi-compartment model of a spinal motoneuron (MN) in XPPAUT and verified its firing accuracy against its original experimental data and against an anatomically detailed cell model that incorporates known MN non-linear firing mechanisms. We used the new model in XPPAUT to study the effects of somatic and dendritic ion channels on the MN bifurcation diagram under normal conditions and after amyotrophic lateral sclerosis (ALS) cellular changes.

**Results:**

Our results show that somatic small-conductance Ca^2+^-activated K (SK) channels and dendritic L-type Ca^2+^ channels have the strongest effects on the bifurcation diagram of MNs under normal conditions. Specifically, somatic SK channels extend the limit cycles and generate a subcritical Hopf bifurcation node in the V-I bifurcation diagram of the MN to replace a supercritical node Hopf node, whereas L-type Ca^2+^ channels shift the limit cycles to negative currents. In ALS, our results show that dendritic enlargement has opposing effects on MN excitability, has a greater overall impact than somatic enlargement, and dendritic overbranching offsets the dendritic enlargement hyperexcitability effects.

**Discussion:**

Together, the new multi-compartment model developed in XPPAUT facilitates studying neuronal excitability in health and disease using bifurcation analysis.

## 1. Introduction

Bifurcation analysis of neural models is a useful tool for studying the steady-state, non-linear dynamics of neurons and the characteristics of their firing output throughout the stimulus input range. While bifurcation analysis has greatly contributed to our understanding of the neuronal non-linear dynamics underlying membrane oscillations and cell firing under normal (Jalics et al., 2010; Zhou et al., 2018), pathological (Glass and Mackey, 1979; Kaper et al., 2013), and pharmacological (V-Ghaffari et al., 2017) conditions, their use has been limited to single-compartment models (Jalics et al., 2010; Wang et al., 2019; Xing et al., 2022). However, such models lack the non-linear behaviors that arise from dendritic channels (Hounsgaard and Kiehn, 1993; Lee and Heckman, 1999; Li and Bennett, 2007; Carlin et al., 2009). While AUTO (Doedel et al., 2007) and MatCont (Dhooge et al., 2003) are the primary bifurcation tools used in the dynamical systems literature, XPPAUT and its integrated module AUTO (Ermentrout and Mahajan, 2003) are the more common bifurcation analysis software used in the neuroscience literature. Because the number of ordinary differential equations of a high-fidelity neuronal model with multi-compartments and multiple ion channels could be large and XPPAUT—unlike NEURON—lacks tools that abstract the mathematical detail to facilitate model development, the process of developing a multi-compartment model in XPPAUT is cumbersome (N.B., no single multi-compartment model of a neuron has been developed in XPPAUT to date). Thus, extending bifurcation analysis to high-fidelity models with multiple compartments and dendritic channels, which mediate many non-linear firing behaviors in MNs, has been limited. As multi-compartment models are more accurate in simulating the firing behaviors of spinal MNs than reduced models (Hendrickson et al., 2011; Elbasiouny, 2014), bifurcation analysis of more complex models and their behaviors is, therefore, of great importance.

The goals of this study are to (1) develop a multi-compartment computer model that simulates in XPPAUT the non-linear behaviors as empirically measured in spinal MNs, and (2) use the model in XPPAUT to assess the role of somatic and dendritic ion channels in regulating the cell’s repetitive firing using bifurcation analysis under normal and disease conditions. As XPPAUT does not support large cell models with 3D anatomical detail, our first step was to reduce the cell model published by Mousa and Elbasiouny (2020) while preserving as much accuracy in simulating this MN’s firing behaviors as possible. The outcome of this step was a six-compartment (6C) model developed in XPPAUT, which we assessed to verify its electrical properties versus experimental data. To the authors’ best knowledge, this model is the *first* multi-compartment neuron model with somatic and dendritic channels developed in XPPAUT.

Our results showed that, under normal conditions, somatic small-conductance calcium-activated potassium (SK) channels, which mediate the afterhyperpolarization (AHP) phase of the action potential (AP), support the MN in generating healthy, large-amplitude AP spikes and stable rhythmic cell firing over an extended input current range. Importantly, the presence of somatic SK channels resulted in the emergence of a subcritical Hopf bifurcation node to replace the supercritical Hopf node at the end of the voltage-current (V-I) bifurcation relationship. This bifurcation point results from the negative feedback control between the somatic SK and N-type Ca^2+^ (CaN) channels. Our results also showed that dendritic L-type Ca^2+^ channels increase the cell excitability substantially and shift stable rhythmic firing on the V-I bifurcation relationship to negative currents, allowing the cell to fire continuously in absence of excitatory stimuli. However, dendritic SK channels regulated L-type Ca^2+^ channels activation toward normal levels, yet continued to enable rhythmic cell firing starting at low input currents. Using the new MN model in XPPAUT to study the impact that somatic hypertrophy and dendritic enlargement and overbranching have on cell firing in ALS, our bifurcation analysis showed that the dendritic enlargement has more notable net hypoexcitability effects on cell firing than does somatic enlargement. Interestingly, some hyperexcitability effects also arose from dendritic enlargement, however dendritic overbranching appeared to offset those effects. Together, this work reports the first multi-compartment MN model developed in XPPAUT and makes it available for the scientific community to use. Additionally, our results report the first bifurcation analysis conducted on a neuron model with this higher level of anatomical and ion channel detail under normal and disease conditions. Further, our bifurcation analysis describes novel non-linear behaviors, as well as the roles different ion channels play in regulating cell firing.

## 2. Materials and methods

In the present study, we used a fatigue-resistant (FR) cat MN model of the medial gastrocnemius (MG) muscle, which was modeled in high detail in Mousa and Elbasiouny (2020). This high-fidelity model incorporates known non-linear firing behaviors of MNs. This model was developed in the NEURON software (Hines and Carnevale, 1997), which does not feature bifurcation analysis. As XPPAUT (Ermentrout and Mahajan, 2003), and its bifurcation analysis AUTO module, do not have tools to facilitate the development of high-fidelity neuronal models with detailed anatomy and multiple ion channels, bifurcation analysis of the full 3D model of Mousa and Elbasiouny (2020) is infeasible, due to the hundreds of balance and ordinary differential equations to be written in XPPAUT. To overcome that, the first goal of the present study was to reduce the 3D Mousa and Elbasiouny (2020) model into a simpler multi-compartment model with all non-linear MN behaviors retained and implemented in XPPAUT, where bifurcation analysis is possible. As Auto-07p usage in neuroscience is still limited and XPPAUT’s time domain simulations were needed for the model verification process and comparison against the NEURON simulations, we used XPPAUT (version 8.0) and AUTO 2000 in our simulations. All figures were constructed using Python3 (Van Rossum and Drake, 2010), Matplotlib (Hunter, 2007), Seaborn (Waskom, 2021), and Pandas (The Pandas Development Team, 2022).

### 2.1. Model description

**FIGURE 1:**
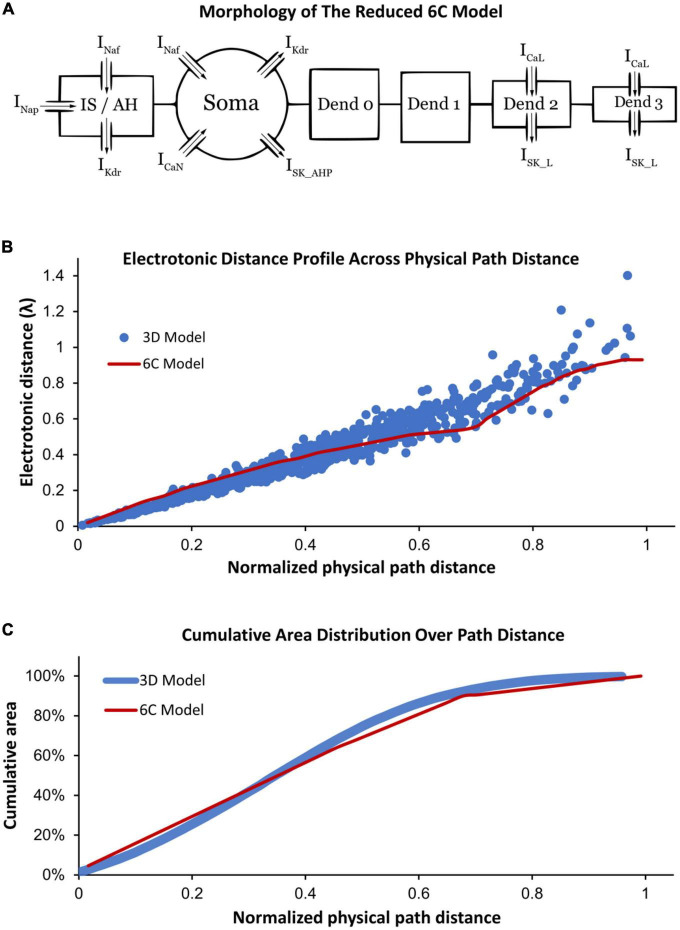
**(A)** Morphology of the reduced six-compartment (6C) model in XPPAUT showing its compartments with their ion channels. The arrows refer to the current direction of each ion channel. **(B)** A comparison between the relationship of the physical path distance and electrotonic distance (in λ) in the reduced 6C model in XPPAUT (red) vs. the 3D model of Mousa and Elbasiouny (2020) (blue). **(C)** A comparison between the cumulative area and physical path distance of the model compartments in the reduced 6C model in XPPAUT (red) and the 3D model of Mousa and Elbasiouny (2020) (blue). In panels **(B,C)**, the *x*-axes are normalized to the longest physical path distance of each model.

Following the methodology of Elbasiouny (2014) in reducing 3D models into reduced models that still demonstrate highly accurate firing behaviors, we reduced the high-fidelity computer model of Mousa and Elbasiouny (2020) into a six-compartment (6C) model (Figure 1A, 6C model morphology, and electrical parameters are shown in Tables 1, 2, respectively). This 6C model was able to simulate experimental data and the 3D model behaviors with acceptable accuracy (Table 3). In the reduction process, the simplified 6C model was designed to resemble the 3D model in area distribution (Figure 1C), electrical distance distribution (Figure 1B), and electrical properties, which were optimized to fall within the 95% confidence interval of experimental data [passive and active membrane properties, rheobase, action potential (AP) and afterhyperpolarization (AHP) properties, frequency-current (FI) relationship properties] and to match the 3D model properties as much as possible.

**TABLE 1:** Geometric properties of the (6C) reduced control model.

Compartment	Length (μm)	Diameter (μm)
Axon hillock	20	12.94
Soma	48.8	48.8
Dendrite 0	1450.97	42.21
Dendrite 1	1450.97	42.21
Dendrite 2	754.84	36.42
Dendrite 3	1037.91	10.19

**TABLE 2:** Channels conductances of the (6C) reduced control model.

Compartment	Ion channel	Channel conductance (siemens/cm^2^)
Initial segment/axon hillock (IS/AH)	Leak	1/225
	Kdr	0.16552
	Naf	1.3392
	Nap	3.2971e-5
Soma	leak	1/225
	Naf	0.06
	Kdr	0.80048
	CaN	0.01
	SK_AHP	0.0221
All dendrites	Leak	1/11000
Dendrite 2	CaL	0.000168
	SK_L	0.00006888
Dendrite 3	CaL	0.000042
	SK_L	0.00001722

**TABLE 3:** Comparison between the 3D and XPPAUT models’ electrical properties vs. experimental data.

Category	Property	Experimental data Mean ± std (95% CI range)	3D model*	6C XPPAUT model
Passive properties	Resting membrane potential (mV)	−70 ± 7 (−73.03, −66.97) (Hochman and McCrea, 1994a)	−70	−70
	Input resistance (MΩ)	1.4 (Fleshman et al., 1988)	1.35	1.39
	Input conductance (μS)	0.8 ± 0.3 (0.69, 0.91) (Manuel, 2005)	0.74	0.72
Spike initiation	Rheobase (nA)	11.0 ± 6.08 (8.97, 13.03) (Foehring et al., 1986)	9	9.1
Action potential (AP)	AP height (mV)	81.8 ± 9.3 (77.78, 85.82) (Hochman and McCrea, 1994b)	79.54	85.32
Afterhyperpolarization (AHP)	AHP depth (mV)	3.13 ± 1.15 (2.63, 3.63) (Hochman and McCrea, 1994b)	2.59	2.92
	AHP ½ decay (ms)	22 ± 5.57 (19.96, 24.04) (Zengel et al., 1985) 22.1 ± 8.5 (20.03, 24.17) (Hochman and McCrea, 1994a)	20.63	23.4
	AHP duration (ms)	81.9 ± 17.1 (74.51, 89.29) (Hochman and McCrea, 1994b) 78 ± 22 (69.83, 86.17) (Zengel et al., 1985)	75.15	80.5
Frequency-current (FI) relationship	Gain (Hz/nA)	1.7 ± 0.5 (1.32, 2.08) (Kernell, 1965)	2.21	1.7
Ca^2+^ PIC	Initial peak (nA)	8.4 ± 7.9 (4.47, 12.33) (Lee and Heckman, 1999)	11.87	12.29
	Onset potential (mV)	−46.5 ± 5.1 (−49.04, −43.96) (Lee and Heckman, 1999)	−49.03	−48.5
	Offset potential (mV)	−57.3 ± 8.4 (−61.48, −53.12) (Lee and Heckman, 1999)	−59.09	−59.35
	ΔV	10.9 ± 6.3 (7.77, 14.03) (Lee and Heckman, 1999)	10.05	10.85
	ΔI	1.6 ± 4.1 (−0.44, 3.64) (Lee and Heckman, 1999)	−0.07	0.06
	SK to L-type Ca^2+^ current ratio	15–26% (Li and Bennett, 2007)	22%	22%

*Mousa and Elbasiouny (2020).

Similar to the 3D model of Mousa and Elbasiouny (2020), the 6C model was formed of initial segment/axon hillock (IS/AH), soma, and four dendrites (Figure 1A). All compartments of the 6C model had passive leak channels. The soma had the following additional channels: fast sodium (Naf), delayed rectifier potassium (Kdr), N-type calcium (CaN), and small conductance calcium-activated potassium (SK_AHP) channels. The IS/AH had the following channels (in addition to leak channels): Naf, persistent sodium (Nap), and Kdr. Dendrites 0 and 1 had only leak channels, whereas dendrites 2 and 3 also contained L-type Ca^2+^ (CaL) and small conductance calcium-activated potassium (SK_L) channels. Importantly, the location and conductance of dendritic CaL and SK_L channels were retained in the 6C model, as in the 3D model of Mousa and Elbasiouny (2020), to generate comparable Ca persistent inward currents (Ca^2+^ PIC). Specifically, the 3D model had dendritic regions of high (26% of the dendritic area located between 0.44λ and 0.6λ from the soma) and low (10% of the dendritic area located between 0.6λ and 1.1λ from the soma) Ca^2+^ PIC and SK conductances. To mimic that, the distal dendrites of the 6C model were simulated with two compartments, one with high Ca^2+^ PIC and SK conductances (dendrite 2, with 26% of the dendritic surface area and distance between 0.45λ and 0.55λ from the soma) and another with low Ca^2+^ PIC and SK conductances (dendrite 3, with 10% of the dendritic surface area and distance between 0.55λ and 0.93λ from the soma). In this way, the reduced 6C model had dendritic conductances very similar to those of the 3D model of Mousa and Elbasiouny (2020).

All ion channels were conductance-based, following the Hodgkin and Huxley formalism (Hodgkin and Huxley, 1952), such that activation and inactivation states are voltage-dependent, except for SK channels which use saturation function that depends on the calcium concentration in the compartment (Mousa and Elbasiouny, 2020). The cell membrane specific capacitance was 1 μF/cm^2^. All model equations are listed in the Supplementary material.

### 2.2. Bifurcation diagrams

In this paper, the V-I bifurcation curves have four colors: (1) red traces indicate stable equilibrium points, (2) black traces indicate unstable equilibrium points, (3) green traces indicate stable periodic solutions; the maximal and minimal value of the green curve at a single current value refers to the maximal and minimal amplitude of the stable oscillation at this current value, and (4) blue traces indicate unstable periodic solutions. In the bifurcation diagram, the firing range is the range where stable periodic solutions exist.

### 2.3. XPPAUT simulation settings

We used the following parameters in XPPAUT: dt is 0.025 ms, meth = backeul, parmax = 1, parmin = −1, NPR = 100,000, NMAX = 10,000,000, NTST = 100. Regarding parmax and parmin, the maximal injected current during all simulations is 50 nA, so a scaling factor is used to map the current range to the parameter range. NTST can be higher in some simulations to avoid mx (failed to converge) error in XPPAUT. The simulation ran using a personal computer with 8 Gb RAM, Intel Core i7-5500 U CPU, and Ubuntu 20.04 OS. Long pulses are generated using unit step pulses, and FI curves are generated using ramp pulses with a slope of 4 nA/s and a maximal current injection of 25 nA.

## 3. Results

### 3.1. Development and verification of the MN model in XPPAUT

To study the role of different ion channels in regulating MN repetitive firing, bifurcation analysis of a MN model with sufficient anatomical and ion channel details in XPPAUT was needed. As XPPAUT does not facilitate simulations of large cell models with 3D anatomical detail, the first goal of this study was to reduce the MN model published in Mousa and Elbasiouny (2020) into an equivalent one that could be implemented in XPPAUT for bifurcation analysis. The Mousa and Elbasiouny (2020) model was chosen as the reference 3D model because it is currently the cell model that simulates the most non-linear electrical and firing behaviors of spinal MNs (e.g., firing bistability, voltage hysteresis, synaptic amplification, PIC warm-up, dendritic staircase currents, dendritic persistent inward and outward currents). We reduced the 3D, 731-compartment anatomy of the Mousa and Elbasiouny (2020) model into an equivalent 6-compartment (6C) model that could be implemented in XPPAUT (Figure 1A). Six were the fewest compartments that could match most of the 3D model’s electrical properties within the 95% confidence interval of experimental data (see Table 3). The 6C model’s morphological parameters were set to replicate the 3D model dendritic area spatial distribution, such that (1) distal dendrites contain CaL and SK_L channels that mediate the Ca^2+^ PIC (see section “Materials and methods” for detail), (2) Electrotonic distance profile matches the original 3D model (Figure 1B), and (3) the cumulative distribution of the dendritic surface area matches the experimental 3D morphology of Cullheim et al. (1987) model (Figure 1C). Also, all channel kinetic parameters were preserved and translated into the XPPAUT environment to develop a 25-state-variable model. To confirm that the 6C MN model was implemented successfully in XPPAUT, a NEURON version of the 6C reduced model was also implemented and the results of both were compared and confirmed to be identical. The 6C model in XPPAUT was then used for bifurcation analysis using the Auto module. With somatic/axon hillock/dendritic compartments, 25 state variables and multiple ion channels, this cell model is the most comprehensive model implemented in XPPAUT to date. This enabled us to perform bifurcation analysis of one neuron with unprecedented details. The following sections present the bifurcation analyses of the 6C model in XPPAUT, in which we added one compartment and one ion channel at a time to examine the effect of each channel on cell firing.

### 3.2. Somatic SK channels support stable rhythmic cell firing over extended input range

**FIGURE 2:**
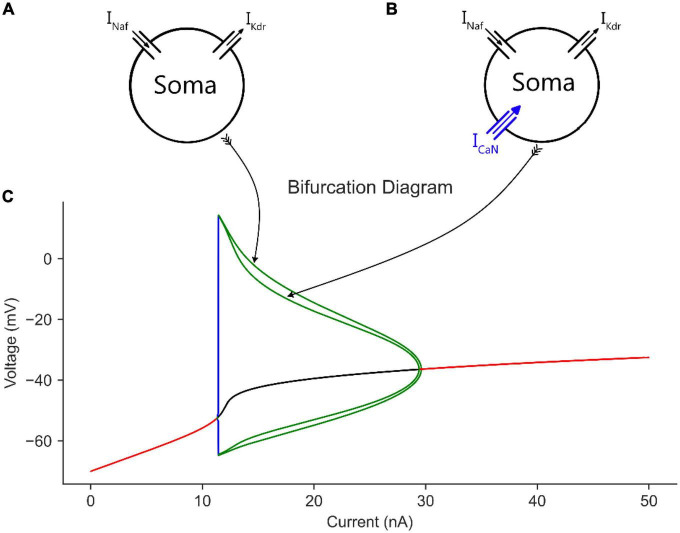
The effect of somatic CaN channels on the MN model bifurcation diagram. **(A)** The soma model with leak, Na, and K channels only. **(B)** The soma model in A with CaN channels added. **(C)** The bifurcation diagram of the soma models with and without CaN channels.

To examine the effect of somatic ion channels on cell firing, we conducted the first bifurcation analysis on a single compartment model composed of only the soma of the 6C model in XPPAUT, with only its somatic leak, Na, and K channels included (Figure 2A). These are the minimal channels needed for generating an AP. We started with this single compartment soma model to serve as a comparison of the following bifurcation graphs, in which additional channels are added to the model one at a time. When bifurcation analysis of the single compartment soma model was conducted, the typical V-I bifurcation graph of a Hodgkin–Huxley model with a firing behavior starting and ending between subcritical and supercritical Hopf bifurcation points, respectively, was obtained (Figure 2C). In this bifurcation diagram, the subthreshold membrane depolarization with no cell firing at low input current is shown by the increasing red trace on the left until rhythmic cell firing is evoked at the cell rheobase (shown by the green traces). As Na channels inactivate gradually with increasing input current, the height of AP decreases gradually (shown by the decreasing limit cycles voltage amplitude in Figure 2C) until cell firing dampens completely (shown by the red trace on the right in Figure 2C).

**FIGURE 3:**
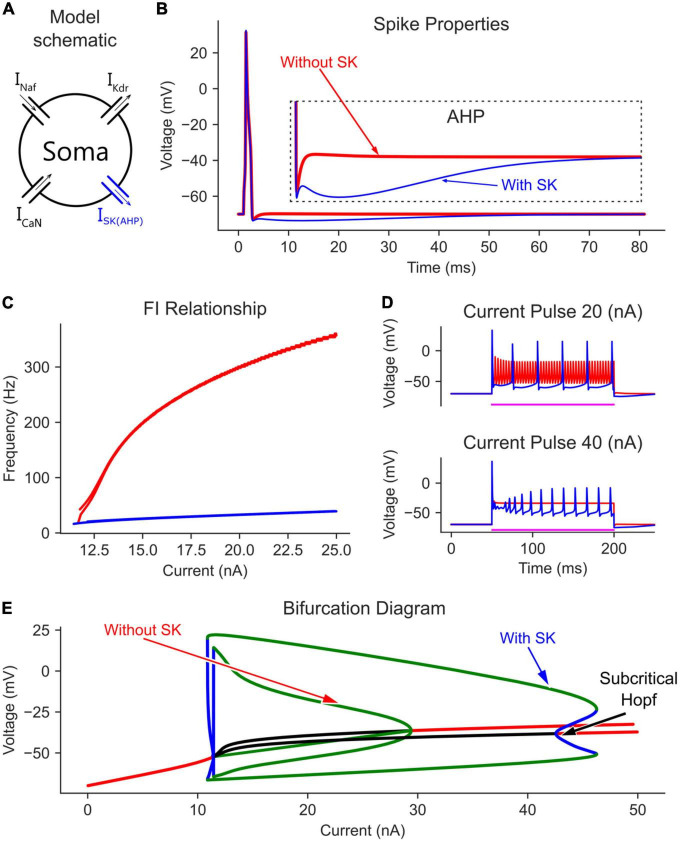
The effect of somatic SK channels on the MN model bifurcation diagram. **(A)** The soma model with SK channels added to the Na, K, and CaN channels. The AP and AHP shapes **(B)**, F-I relationship **(C),** and MN repetitive firing on long pulses **(D)** with (blue traces) and without (red traces) the somatic SK channels included in the model. **(E)** The V-I bifurcation diagram of the MN model with and without the somatic SK channels. In the diagram, the green and blue traces indicate the extrema of the stable and unstable limit cycles, respectively. The red and black traces show stable and unstable equilibrium points, respectively.

When CaN channels were added to the soma model (Figure 2B), a similar voltage amplitude (but with a narrower limit cycles’ range) and a bit smaller current range were seen in the bifurcation graph (Figure 2C). The decrease in limit cycles voltage amplitude and current range is due to the additional depolarization provided by the CaN channels, which reduced the AP amplitude more. However, when the somatic SK channels were next added to the soma model (Figure 3A), which mediate the spike AHP (Figure 3B, blue traces), the limit cycles voltage amplitude and current range increased substantially, and the supercritical Hopf node at the end of the firing range was replaced with a subcritical node (Figure 3E, the “with SK” trace). Given the negative feedback control exerted on the membrane potential by the somatic SK and N-type Ca (CaN) channels, the subcritical bifurcation node in the V-I bifurcation graph reflects this membrane potential non-linearity.

In the time domain, the negative feedback control provided by somatic SK channels on the membrane potential was evident. For instance, without somatic SK channels included, the frequency-current (F-I) relationship in response to a triangular current ramp reached very high, non-physiological firing rates, and had a steep slope (Figure 3C, red trace). Also, long current pulses evoked dwarf APs firing at a high rate (Figure 3D, red trace). However, the addition of SK channels regulated the firing of the neuron model and brought the F-I relationship slope and long pulse firing down to the physiological firing range (blue traces in Figures 3C, D). Interestingly, at 40 nA, the model without SK channels had fully inactivated Na channels with no cell firing (Figure 3D, lower panel). However, the AHP mediated by SK channels helped relieve some Na channels from inactivation and evoked cell firing, which is also reflected in the bifurcation diagram at 40 nA (Figure 3E, there are no limit cycles for the model without SK at 40 nA). Collectively, somatic SK channels support the generation of full (large amplitude) and stable rhythmic cell firing over an extended input current range and generate a subcritical Hopf bifurcation point in the V-I bifurcation graph.

### 3.3. Dendritic L-type Ca^2+^ channels enable MN firing at lower currents

**FIGURE 4:**
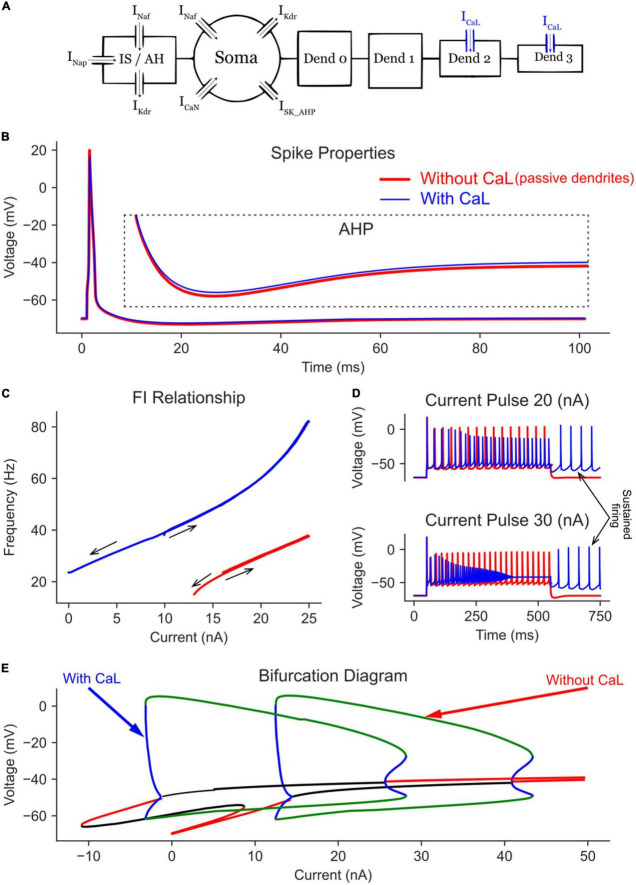
The effect of dendritic L-type Ca channels on the MN model bifurcation diagram. **(A)** The 6C model morphology and the ion channels involved. Note that all compartments have leak channels, but CaL channels are on dend 2 and 3 only. The AP and AHP shapes **(B)**, F-I relationship **(C),** and MN repetitive firing on long pulses **(D)** with (blue traces) and without (red traces) the dendritic L-type Ca channels included in the model. **(E)** The V-I bifurcation diagram of the MN model with and without the dendritic L-type Ca channels. In the diagram, the green and blue traces indicate the extrema of the stable and unstable limit cycles, respectively. The red and black traces show stable and unstable equilibrium points, respectively.

According to Li and Bennett (2007), L-type Ca^2+^ and SK channels are present on the dendrites of MNs, which were simulated in Mousa and Elbasiouny (2020). The currents of both channels summate to produce Ca^2+^ persistent inward currents (Ca^2+^ PICs). In this section, we first investigate the effects of the L-type Ca^2+^ channels alone. To that end, we added four dendritic compartments to the somatic section to form the complete 6C model (Figure 4A), then added L-type Ca^2+^ channels to the distal dendrites (dendrites 2 and 3 in Figure 4A) to replicate the 3D model of Mousa and Elbasiouny (2020). The somatic spike and AHP of the complete 6C model before and after adding the dendritic L-type Ca2^+^ channels are shown in Figure 4B. Without L-type Ca^2+^ channels, the model with passive dendrites showed a similar bifurcation diagram to the one with only a somatic compartment (compare the “with SK” trace in Figure 3E to the “Without CaL” trace in Figure 4E). However, the limit cycles were a little shortened in both voltage and current ranges, as the somatic current now activates a larger cell area with larger capacitance, which in turn causes a smaller AP height. The firing behavior during the current ramp (F-I relationship) was similar to that of the somatic compartment model, but with lower firing rates due to the larger cell surface area and capacitance.

When L-type Ca^2+^ channels were added to the passive dendrites (Figure 4E), the limit cycles were shifted in the negative current direction (by > 12 nA), allowing the cell to fire repetitively at zero current (i.e., without input). This behavior was also seen in the F-I relationship, where the cell continued to fire on the descending current ramp until zero current was injected (Figure 4C). Long pulse simulations showed similar results, where the cell continued to fire repetitively after the current pulse was terminated (Figure 4D, see the arrow showing self-sustained firing). The addition of L-type Ca^2+^ channels increased the MN maximal firing rate from 37.8 to 82.4 Hz on the current ramp (Figure 4C) and increased the average firing rate during long pulses (Figure 4D).

The bifurcation analysis shows that, at low current amplitudes (< 10 nA), two stable states exist in the MN model with dendritic Ca channels (Figure 4E, the “with CaL” trace): one non-oscillatory state representing the subthreshold membrane depolarization (the red line) and another oscillating state with limit cycles representing the cell firing (between the green lines). The presence of these two stable states indicates that a MN with dendritic Ca^2+^ PIC may fire or not depending on its initial conditions, which is reflected in the F-I relationship (Figure 4C, blue trace) with no cell firing in the range < 10 nA on the ascending firing segment but with repetitive cell firing in the same range during the descending firing segment. The temporal activation of Ca^2+^ PIC is seen during the long pulse simulations, in which a 30 nA current pulse evoked repetitive cell firing at the beginning of the pulse; then it changed to a non-oscillatory steady state membrane depolarization at the end of the pulse (due to full Na channels inactivation). In sum, L-type Ca^2+^ channels shift in the MN bifurcation diagram to lower currents, allowing the cell to fire at negative currents (i.e., in absence of excitation) and at higher firing rates.

### 3.4. Dendritic SK channels regulate L-type Ca^2+^ channels activation

**FIGURE 5:**
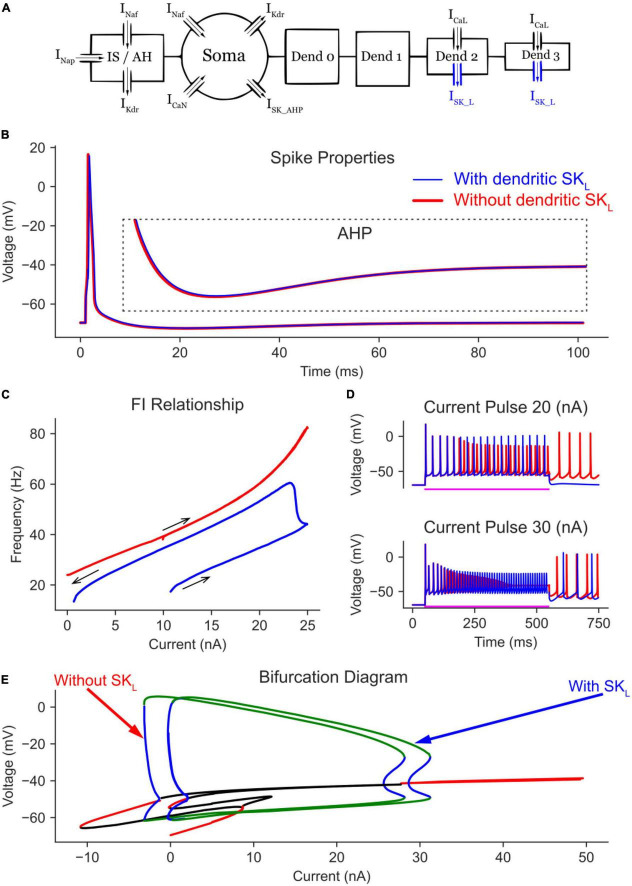
The effect of dendritic SK_L channels on the MN model bifurcation diagram. **(A)** The 6C model morphology and the ion channels involved. Note the addition of SK channels to dendrites 2 and 3. The AP and AHP shapes **(B)**, F-I relationship **(C)**, and MN repetitive firing on long pulses **(D)** with (blue traces) and without (red traces) the dendritic SK channels included in the model. **(E)** The V-I bifurcation diagram of the MN model with and without the dendritic SK channels. In the diagram, the green and blue traces indicate the extrema of the stable and unstable limit cycles, respectively. The red and black traces show stable and unstable equilibrium points, respectively.

Dendritic L-type Ca^2+^ channels co-localize with SK channels on the dendrites to regulate the amplitude of Ca^2+^ PIC (Li and Bennett, 2007). When SK channels were added to the model’s distal dendrites (Figure 5A), the bifurcation diagram shifted to the right, as it partially deactivated L-type Ca^2+^ channels (i.e., reduced the Ca^2+^ PIC) and stopped cell firing at less negative current (Figure 5E). This was observed in the F-I relationship (Figure 5C, blue trace), in which dendritic SK channels allowed graded activation of the Ca^2+^ PIC, causing the cell to fire at lower firing rates and for bistability to emerge (i.e., non-overlapping ascending and descending F-I segments in Figure 5C, blue trace). In absence of dendritic SK channels, L-type Ca^2+^ channels fully activate immediately, leading to Ca^2+^ PIC saturation upon cell recruitment and lack of firing bistability (i.e., overlapping ascending and descending F-I segments) (Figure 5C, red trace). The effect of dendritic SK channels is primarily on repetitive firing as they had negligible effect on single spike properties (Figure 5B).

The effects of graded Ca^2+^ PIC activation by dendritic SK channels were seen in the time domain long pulse simulations (Figure 5D, blue traces) which show no self-sustained firing after the termination of a 20 nA current pulse (as L-type Ca^2+^ channels were not fully activated), but with self-sustained firing present after the termination of a 30 nA current pulse (as L-type Ca^2+^ channels were fully activated by the very high amplitude pulse). In absence of dendritic SK channels, self-sustained firing was always present regardless of pulse amplitude (Figure 5D, red traces). Interestingly, and contrary to their somatic counterpart, dendritic SK channels did not extend the limit cycles or the cell firing range (as in Figure 3E). They only shifted the bifurcation diagram rightward, offsetting some of the Ca^2+^ PIC effects.

### 3.5. Examining mechanisms of MN excitability dysfunction in ALS

**FIGURE 6:**
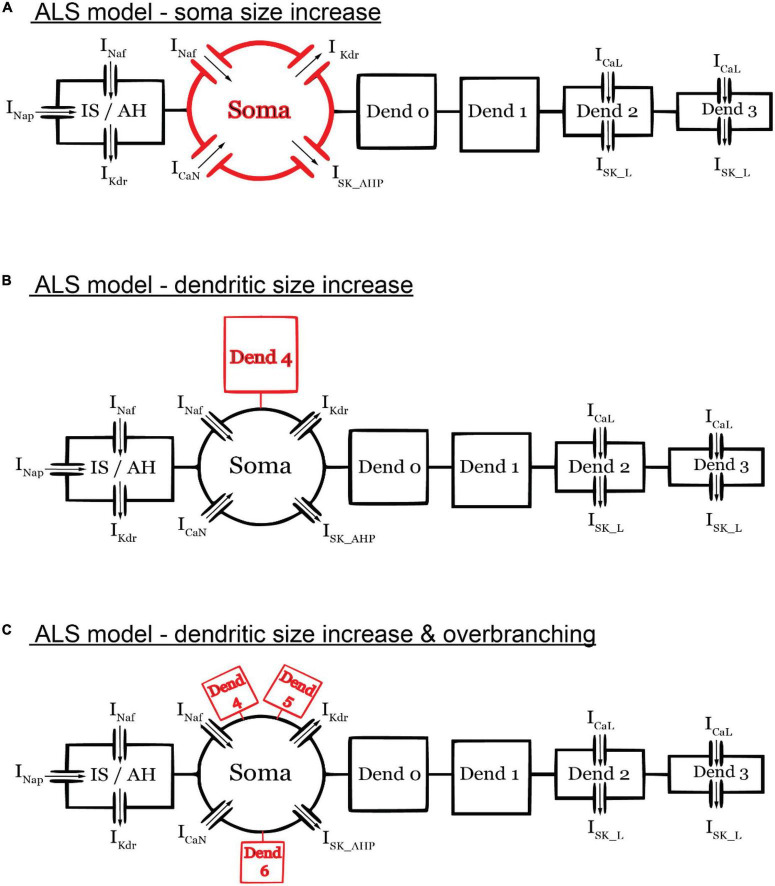
Schematic diagrams of the ALS models. **(A)** ALS model with only soma size increased (by 10%). **(B)** ALS model with only dendritic size increase (by 30%) *via* the addition of one dendritic branch. **(C)** ALS model with only dendritic size increase (by 30%) *via* the addition of three dendritic branches. The diagrams are not to scale. All other model parameters were unchanged in the ALS models.

To illustrate the utility of the 6C model in XPPAUT in studying neuronal excitability dysfunction in neurodegenerative diseases, we examined the effect of pathological MN anatomical changes in ALS by conducting bifurcation analysis on the 6C model in XPPAUT under ALS conditions. Specifically, in the early stages of ALS, spinal MNs undergo anatomical changes, such as somatic (Shoenfeld et al., 2014; Dukkipati et al., 2018) and dendritic enlargement (Amendola and Durand, 2008), as well as dendritic overbranching (Filipchuk and Durand, 2012) in multiple ALS mouse models. While these individual cellular changes are expected to cause cell hypoexcitability, it is unknown whether these changes are of similar magnitude and impact on MN excitability. To examine the effects of somatic vs. dendritic size enlargement, we increased the soma size by 10% [based on experimental data of P10 SOD MN increases of 6–20%, (Dukkipati et al., 2018; Figure 6A)] and compared that to a 30% increase in the dendritic size [based on experimental data of P10 SOD MN increases of 25–90%, (Amendola and Durand, 2008)] *via* the addition of one dendritic branch (Figure 6B). In these simulations, all other model parameters, including the ionic conductances, were unchanged.

**FIGURE 7:**
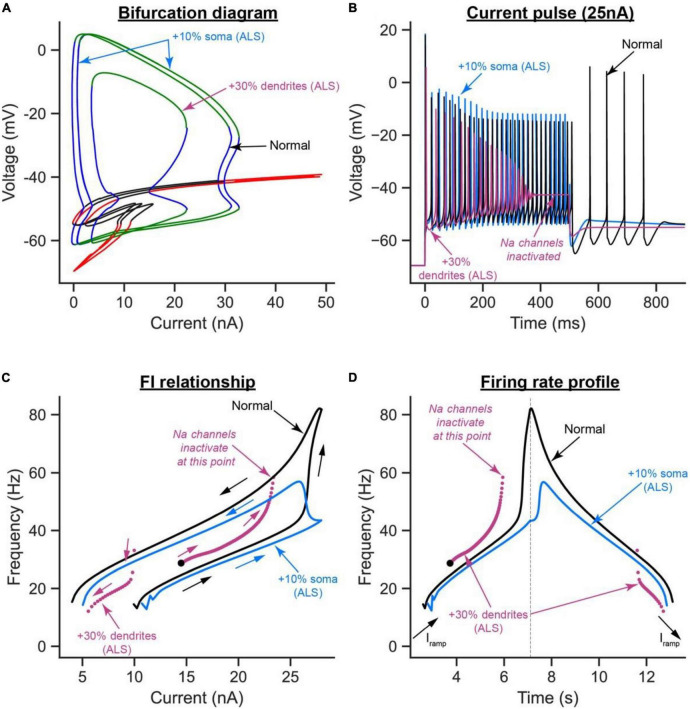
The effects of somatic vs. dendritic size enlargement in ALS on MN firing. **(A)** The V-I bifurcation diagrams, **(B)** cell firing on a 25 nA long current pulse, **(C)** FI relationships during ramp current injection, and **(D)** firing rate profiles of the control model (normal condition, black traces) compared to the ALS model with the somatic area increased by 10% (blue traces) or the ALS model with the dendritic area increased by 30% (purple traces).

Interestingly, relative to the normal model, the increase in soma size shifted the bifurcation diagram to the right, causing the cell recruitment current to increase and the limit cycles’ height to also increase at any given current (Figure 7A, blue label). These hypoexcitability effects were confirmed in the time domain, as the AP height was increased and self-sustained firing was absent (Figure 7B, blue labels), recruitment current was increased, and firing bistability (measured as the difference between the ascending and descending FI relationship segments) was decreased (Figure 7C, blue labels), and cell firing rates were generally decreased (Figures 7C, D, blue labels).

Conversely, the increase in dendritic area had mixed effects on the cell excitability. For instance, relative to the normal model, the cell recruitment current was increased, the limit cycles’ height and range were reduced, and the subcritical Hopf bifurcation node in the bifurcation diagram was exacerbated, effectively shrinking the operational firing range of the cell significantly (Figure 7A, purple label). These are hypoexcitability changes whose effects were observed in the time domain as a reduction in the AP height (Figure 7B, purple label), lack of cell ability to fire repetitively due to Na channel inactivation (Figure 7B, purple label), and a significant increase in cell recruitment current (Figure 7C, purple label). Paradoxically, the increase in dendritic area increased the cell’s repetitive firing on the ascending F-I segment relative to the normal model (compare the purple to black segments in the left part of Figure 7D), which is a hyperexcitability change. This increase in ascending firing rates was due to the significant increase in cell capacitance due to the dendritic enlargement, leading to an increase in the cell time constant (i.e., slower activation of the cell); thereby causing Ca^2+^ PIC full activation before cell recruitment, which inactivated Na channels and reduced the cell firing range. Collectively, these results show that somatic and dendritic enlargements have different effects on MN excitability in ALS, with the dendritic enlargement having much stronger hypoexcitability effects on MN excitability than those from soma size increase, primarily because of the significant increase in cell capacitance by the dendritic enlargement.

**FIGURE 8:**
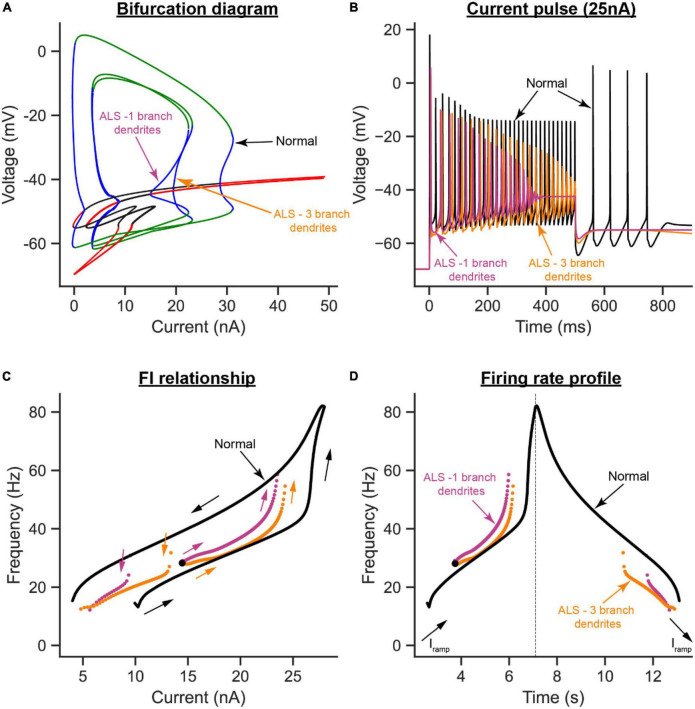
The effects of dendritic overbranching in ALS on MN firing. **(A)** The V-I bifurcation diagrams, **(B)** cell firing on a 25 nA long current pulse, **(C)** F-I relationships during ramp current injection, and **(D)** firing rate profiles of the control model (normal condition, black traces) compared to the ALS models with dendritic area increased by 30% *via* a 1-branch dendrite (purple traces) or a 3-branch dendrite (orange traces).

To examine the effects of dendritic overbranching on cell excitability, we compared the ALS model when the dendritic area was enlarged by 30% *via* one (Figure 6B) vs. three dendritic branches (Figure 6C). Interestingly, although the dendritic area was increased by the same percentage in both ALS models and both models experienced hypoexcitability effects relative to the normal condition, the hypoexcitability effects were less in the model with dendritic overbranching (Figure 8). Specifically, relative to the one-branch ALS model, the three-branch ALS model had larger operational firing range (as the subcritical Hopf node in the bifurcation diagram shifted rightward, Figure 8A—orange label) and repetitive cell firing on long pulses and ramps was maintained longer (Figures 8B, D—orange label) and at less firing rate (Figures 8C, D—orange label). Together, these results show that dendritic overbranching attenuates the hyperexcitability effects of the dendritic enlargement, leading to less firing rates.

## 4. Discussion

The present study has several highly novel aspects. First, it presents the development of the first (to our knowledge) multi-compartment neuron model in XPPAUT (model code is publicly available for researchers to download and use^1^). Second, using this novel model, we were able, for the first time, to study the effects of somatic and dendritic ion channels on the bifurcation diagrams of spinal MNs. Third, our bifurcation analysis reports novel bifurcation behaviors, such as the generation of a new subcritical Hopf bifurcation node, resulting from somatic SK channels. To demonstrate the utility of the novel model in XPPAUT in investigating neurodegenerative diseases, we used the model to study and contrast the separate effects that somatic and dendritic enlargements have on MN excitability in ALS. Our results show that, under normal conditions, somatic SK and dendritic L-type Ca^2+^ channels have the strongest effects on the bifurcation diagram of spinal MNs. Specifically, somatic SK channels extend the limit cycles range and generate a new subcritical Hopf bifurcation node in the V-I bifurcation diagram of the MN. L-type Ca^2+^ channels do not influence the limit cycles’ shape but shift them to negative currents (reflecting self-sustained cell firing in absence of input). Dendritic SK channels—a less powerful player than their somatic counterpart—lessen the Ca^2+^ PIC effects and shift the limit cycles back toward positive currents. Under ALS conditions, our bifurcation analysis shows that, while somatic and dendritic enlargements have net hypoexcitability effects on the cell, those from the dendritic enlargement are more drastic, reduce the cell operational firing range, and induce some hyperexcitability effects. Importantly, dendritic overbranching appeared to offset the dendritic enlargement hyperexcitability effects. On the other hand, somatic enlargement lowers the cell firing without compromising the cell firing range. Accordingly, the novel XPPAUT 6C model has demonstrated the potential to improve our understanding of how pathological changes impact neuronal excitability in neurodegenerative diseases.

### 4.1. Negative feedback loops are different

Neuronal firing is the outcome of many interacting non-linear membrane properties and ionic mechanisms, and bifurcation analysis provides a means to study and quantify the effect(s) each membrane property has on cell firing. As XPPAUT does not have tools to facilitate the development of high-fidelity neuronal models with detailed anatomy and multiple ion channels, bifurcation analysis has been traditionally conducted on reduced neuronal models of a single compartment with only a few ion channels (White et al., 1995; Wang et al., 2019, 2021; Xing et al., 2022). While bifurcation analysis of reduced neuronal models has been instrumental in shaping our understanding of the neuronal non-linear dynamics underlying membrane oscillations and cell firing under normal (Jalics et al., 2010; Zhou et al., 2018), pathological (Glass and Mackey, 1979; Kaper et al., 2013), and pharmacological (V-Ghaffari et al., 2017) conditions, extending this analysis to higher-fidelity models with multiple compartments and dendritic channels, which mediate many non-linear firing behaviors in MNs (Hounsgaard and Kiehn, 1993; Li and Bennett, 2007; Carlin et al., 2009) has been limited. As multi-compartment models are more accurate in simulating the firing behaviors of spinal MNs than reduced models (Hendrickson et al., 2011; Elbasiouny, 2014), bifurcation analysis of their behaviors is, therefore, of a great importance. Accordingly, the multi-compartment model developed in the present study in XPPAUT is a novel tool that allows the scientific community to study the effects that somatic and dendritic ion channels have on the bifurcation diagrams of neurons. Applied on a spinal MN, our bifurcation analysis showed a subcritical Hopf node in the cell V-I bifurcation diagram, resulting from somatic SK channels. A subcritical Hopf node is a bifurcation node in which unstable limit cycles surround a stable equilibrium point. Our analysis shows that the subcritical Hopf node is mediated by somatic SK channels due to its negative feedback effects on the membrane potential. Interestingly, while dendritic SK and L-type Ca^2+^channels have similar negative feedback effects between them, their interaction did not generate a subcritical Hopf node in the bifurcation diagram (no subcritical Hopf bifurcation node emerged when somatic SK channels were deactivated but dendritic SK channels were present, data not shown). However, because CaN channels inactivate whereas L-type Ca^2+^channels do not, the negative feedback loop between the somatic SK and CaN channels is dynamic (i.e., changes in magnitude with time), whereas that between the dendritic SK and L-type Ca^2+^channels is static. This difference in negative feedback loop dynamics is probably responsible for the appearance of the subcritical Hopf node with the somatic, but not dendritic, SK channels. Together, these results indicate that not all negative feedback loops have similar effects on cell firing.

### 4.2. Neuronal input dynamics

Neurons (including MNs and interneurons) receive inputs from presynaptic sources and these synaptic inputs could be constant at times or dynamic at other times. Because bifurcation analysis examines steady state responses of neurons, we tested the model with long current pulses (to evoke steady-state cell firing) to relate the model firing behavior to the bifurcation diagrams (see long pulse simulations in Figures 3D, 4D, 5D, 7B, 8B). To also study dynamic firing of the cell, we tested the MN model with increasing/decreasing triangular current ramps (see current ramp simulations in Figures 3C, 4C, 5C, 7C, D, 8C, D). Therefore, the analysis in the present study covered both constant and dynamic responses of the MN model. While conducted on a MN model, this work could be extended to other types of neurons or interneurons (e.g., central pattern generator interneurons) in the nervous system.

### 4.3. Bifurcation analysis of diseased neurons

In many neurological conditions, neurons typically experience excitability dysfunction to which somatic and dendritic anatomical changes contribute, such as in ALS (Amendola and Durand, 2008; Elbasiouny et al., 2012; Shoenfeld et al., 2014; Dukkipati et al., 2018), spinal cord injury (Kitzman, 2005), and Alzheimer’s disease (Anderton et al., 1998). For bifurcation analysis to examine neuronal excitability dysfunction in these conditions, multi-compartment models that represent dendritic anatomy and ion channels must be developed in XPPAUT. The multi-compartment model in XPPAUT developed in the present study is a first step in that direction. We demonstrated the utility of that model in investigating the roles somatic and dendritic anatomical changes play in MN excitability dysfunction in ALS. One primary result of our work is that the response of the cell to somatic enlargement is very different from that to dendritic enlargement. While both were expected to reduce the excitability of the cell, somatic enlargement increased the cell recruitment current and reduced the cell firing rate without affecting the operational firing range of the cell (i.e., the range of stable limit cycles along the *x*-axis in Figure 7A). Dendritic enlargement, on the other hand, had more drastic hypoexcitability effects on the cell, e.g., increased cell recruitment current (Figures 7A, C), and lowered descending firing rate (Figures 7C, D). Dendritic enlargement also reduced the MN’s operational firing rate substantially (Figure 7A), yet in some respects induced cell hyperexcitability by increasing the cell’s ascending firing rate (Figures 7C, D). This increase in firing rate was due to the increase in cell capacitance because of the dendritic enlargement, leading to increased cell time constant; thereby causing Ca PIC full activation before cell recruitment, which inactivated Na channels and reduced the cell firing range. The mixed dendritic enlargement excitability effects is important as it illustrates how one cellular change could induce both hypo- and hyperexcitability effects on the MN, which is highly relevant to the conflicting literature about MN excitability dysfunction in ALS [for review, see Elbasiouny (2022)]. In our ALS simulations, we tried to keep the percent changes we tested within the range of what has been reported experimentally. For dendritic enlargement, we only tested 30%, whereas the mean of reported data is ∼58%, because the cell stopped firing when the dendritic size was increased by > 40%. This prevented us from conducting bifurcation analysis of the effects that dendritic enlargement has on cell firing. Therefore, our reported effects on dendritic enlargement are likely to be underestimated. When both the soma and dendrites are enlarged, the cell’s intrinsic excitability dropped significantly such that the cell was no longer able to fire action potential (data not shown). Given that it is experimentally challenging to study the separate effects of neuronal anatomical changes, bifurcation analysis is an important tool that fulfills this role, and can provide deep insight on disease pathogenesis.

Another primary result of our work is that dendritic overbranching is a hypoexcitability mechanism in itself that offsets the dendritic enlargement hyperexcitability effects resulting from the cell increased capacitance. As dendritic overbranching leads to the flow of less current through more dendritic branches, this mechanism generates less dendritic membrane depolarization; thereby, less dendritic PIC and firing rate. This result could partially explain why SOD MNs, which experience dendritic enlargement and overbranching, had no increased dendritic Ca transients, relative to WT, despite having larger Ca PIC (Quinlan et al., 2011, 2015). Additionally, this result highlights the importance of simulating dendritic branching in reduced models of MNs, as single-branch models will not simulate the dendritic active properties accurately, as shown in this and earlier studies (Hendrickson et al., 2011; Elbasiouny, 2014). An XPPAUT MN model with multiple dendritic branches, as the one developed in the present study, is therefore an important tool for more accurate bifurcation analysis of MN behaviors.

## 5. Conclusion

In conclusion, the novel multi-compartment MN model developed in XPPAUT in the present study, which is publicly available to the scientific community, is expected to greatly expand our capabilities to study neuronal function under normal and disease conditions.

## Data availability statement

The datasets presented in this study can be found in online repositories. The code of the 6C model developed in XPPAUT for this study can be found at: https://github.com/MuhammadMoustafa/Bifurcation-analysis-of-spinal-motoneuron-firing-behaviour. The names of the repository/repositories and accession number(s) can be found in the article/supplementary material.

## Author contributions

SE conceived the presented idea. MM and MHM conducted the work and wrote the original draft. SE, MHM, MM, MS, and TB discussed the results. MS, TB, and SE supervised the project and wrote, reviewed, and edited the manuscript. All authors have read and agreed to the published version of the manuscript.

## Footnotes

1
https://github.com/MuhammadMoustafa/Bifurcation-analysis-of-spinal-motoneuron-firing-behaviour
