# Supplementary material for: Bifurcation analysis of motoneuronal excitability mechanisms under normal and ALS conditions
Source: Front Cell Neurosci. 2023 Feb 16;17:1093199. doi: 10.3389/fncel.2023.1093199 (PMC9978418; doi:10.3389/fncel.2023.1093199)
Supplement: Supplementary file 1 [file Data_Sheet_1.docx]

Supplementary Material

# Appendix

The ionic currents I_ion_ of different channels can be described by the following general Expression.

|  | $I_{ion}= g_{ion} \times\left( V_{m}- E_{ion} \right)$ | (A1) |
| --- | --- | --- |
|  |  | (A2) |

where *g_ion_* is the varying conductance of the ion channel; is the maximum conductance of the ion channel listed in table 1; *m* and *h* are the activation and inactivation gating variables (states), respectively, ranging between 0 and 1; *n* and *l* are the order of activation and inactivation, respectively.

For each membrane state variable (η), the time and voltage dependence is given by:

|  | ${d\eta}/{dt= \alpha_{\eta}\left( 1-\eta\right)- \beta_{\eta}\eta}$ | (A3) |
| --- | --- | --- |
|  | $\tau_{\eta}=1/(\alpha_{\eta}+\beta_{\eta})$ | (A4) |

**Fast Na+ channels**

|  | $I_{Naf}= \bar{g}_{Naf}\times m^{3}\times h\times(V_{m}- E_{Na})$ | (A5) |
| --- | --- | --- |
|  | $E_{Na}= 50 mV$ | (A6) |
|  | $\alpha_{m}=\left[ -0.4\times\left( V_{m}+49 \right) \right]/[e^{(-(V_{\infty}+49)/5)}-1]$ | (A7) |
|  | $\beta_{m}=\left[ 0.4\times\left( V_{m}+25 \right) \right]/[e^{((V_{m}+25)/5)}-1]$ | (A8) |
|  | $\tau_{h}=30/[e^{((V_{\infty}+60)/15)}+e^{(-(V_{\infty}+60)/16)}]$ | (A9) |
|  | $h_{\infty}=1/[e^{((V_{m}+58)/7)}+1]$ | (A10) |

**Delayed rectifier K+ channels**

|  | $I_{Kdr}= \bar{g}_{Kdr}\times n^{4}\times(V_{m}- E_{ka})$ | (A11) |
| --- | --- | --- |
|  | $E_{K}=-80 mV$ | (A12) |
|  | $\tau_{n}=5/[e^{((V_{\infty}+50)/40)}+e^{(-(V_{\infty}+50)/50)}]$ | (A13) |
|  | $n_{\infty}=1/[e^{((V_{\infty}+31)/-15)}+1]$ | (A14) |

**Calcium dynamics**

The intracellular Ca^2+^ concentration (measured in mM) in the soma depends on the total compartmental Ca^2+^ current, *I_Ca_*:

|  | ${{d\left[ Ca \right]}_{i}}/{dt}=f \times\left[ -\alpha\times I_{Ca}- k_{ca} \times\left[ Ca \right]_{i} \right]$ | (A15) |
| --- | --- | --- |
|  | $f=0.01$ $\alpha=1 mM/mC/{cm}^{2}$ $k_{ca}=8 {ms}^{-1}$ | (A16) |
|  | $\left[ {Ca}^{2+} \right]_{o}=2 mM$ $\left[ {Ca}^{2+} \right]_{i\vert t=0}=0.0001 mM$ | (A17) |

where *f* is the percent of free to bound *Ca^2+^*. The parameter *α* converts the total *ICa* to *Ca^2+^* concentration. *kCa* is the *Ca^2+^* removal rate, where *Ca^2+^* is removed by uptake into internal stores or by pump extrusion. In the soma, the total *Ca^2+^* current is mediated by the N-type *Ca^2+^* channels. [*Ca^2+^*]*o* is the extracellular *Ca^2+^* concentration, and [*Ca^2+^*]*i* ׀*t=*0 is the intracellular *Ca^2+^* concentration at time =0.

**N-type Ca^2+^ channels**

|  | $I_{CaN}= \bar{g}_{CaN}\times m^{2}\times h \times(V_{m}- E_{Ca})$ | (A18) |
| --- | --- | --- |
|  | $\tau_{m}=15 ms$ | (A19) |
|  | $m_{\infty}=1/[e^{((V_{m}+25)/-5)}+1]$ | (A20) |
|  | $\tau_{h}=50 ms$ | (A21) |
|  | $h_{\infty}=1/[e^{((V_{m}+43)/5)}+1]$ | (A22) |

**Somatic small-conductance Ca^2+^-dependent K^+^ channels (SK_AHP_)**

|  | $I_{{SK}_{AHP}}=\bar{g}_{{SK}_{AHP}}\times\left[ \left[ {Ca}_{N}^{2+} \right]_{i}/\left( \left[ {Ca}_{N}^{2+} \right]_{i}+K_{d} \right) \right]\times\left( V_{m}-E_{k} \right)$ | (A23) |
| --- | --- | --- |
|  | $K_{d}=0.0005 mM$ $E_{k}=-80 mV$ | (A24) |

where [${Ca}_{N}^{2+}$]*i* is the concentration of intracellular calcium mediated via the N-type Ca^2+^ channels, and *K_d_* is the half-saturation level.

**Dendritic L-type Ca^2+^ channels**

|  | $I_{L-Ca}= \overline{g}_{L-Ca} \times l \times(V_{m}- E_{Ca})$ | (A25) |
| --- | --- | --- |
|  | $l_{\infty}=1/\left\{ e^{\left[ \left( V_{m}+43 \right)/-6 \right]}+1 \right\}$ | (A26) |
|  | $E_{Ca}=60 mV \tau_{l (act)}=10 ms \tau_{l (deact)}=60 ms$ $\overline{g}_{L-Ca}=see table 2$ | (A27) |

**Dendritic small-conductance Ca^2+^-dependent K^+^ (SK_L_) channels**

|  | $I_{{SK}_{L}}=\bar{g}_{{SK}_{L}}\times S \times\left( V_{m}-E_{k} \right)$ | (A28) |
| --- | --- | --- |

SK_L_ channels get activated according to the following hill equation

|  | $S_{\infty}=1/\left\{ 1+\left[ K_{d}/\left[ {Ca}_{L}^{2+} \right]_{i} \right]^{H} \right\}$ | | (A29) |  |
| --- | --- | --- | --- | --- |
|  | $\tau_{S}=40 ms$ $K_{d}=0.0005 mM$ $E_{k}=-80 mV H=10$ | (A30) | | |
